# The Effect of Timeliness of Care on Lung Cancer Survival – A Population-Based Approach

**DOI:** 10.5334/aogh.3845

**Published:** 2023-06-05

**Authors:** Teresa Guerreiro, Alexandra Mayer, Pedro Aguiar, António Araújo, Carla Nunes

**Affiliations:** 1 NOVA National School of Public Health, NOVA University of Lisbon, Portugal; 2 Portuguese Institute of Oncology Francisco Gentil, Lisbon, Portugal; 3 Public Health Research Center, NOVA University of Lisbon, Portugal; 4 CHUPorto –University Hospitalar Center of Porto, Porto, Portugal; 5 UMIB-Unit for Multidisciplinary Research in Biomedicine, ICBAS - School of Medicine and Biomedical Sciences, University of Porto, Porto, Portugal

**Keywords:** lung cancer, survival, quality of care, treatment timeliness

## Abstract

**Background:**

Timeliness of care is an important dimension of healthcare quality but it’s unclear whether it improves clinical outcomes in lung cancer (LC) patients.

**Objectives:**

This study aims to analyze treatment patterns, time-to-treatment (TTT) and the impact of treatment timeliness (TT) in overall survival (OS) of patients diagnosed with LC in 2009–2014 in a population-based registry from Southern Portugal.

**Materials and Methods:**

We estimated median TTT for overall population, treatment type and stage. The impact of treatment and TT on five-year OS was analyzed using the Kaplan-Meier method and Cox regression modelling to determine the hazard ratio (HR) of death associated with treatment and TT.

**Results:**

From the 11,308 cases diagnosed, 61.7% received treatment. Treatment rate decreased with increasing stage from 88% in stage I to 66.1% in stage IV. Overall median TTT was 49 days (IQR: 28–88) and 43.3% received TT. Surgery had a longer TTT than radiotherapy and systemic treatment. Patients in earlier stages had lower TT rates and longer TTT compared to more advanced, 24.7% and 80 days in stage I versus 51.3% and 42 days in stage IV (p < 0.0001). OS was 14.9% for total population and 19.6% and 7.1% for patients with and without treatment registered, respectively. TT had no observed impact on OS for stages I/II but a negative effect for stages III/IV. Relative to treated, the adjusted mortality risk was higher in untreated patients (HR = 2.240; 95%CI: 2.293–2.553). Contrary to treatment, TT had a negative impact on survival, with 11.3% in timely vs. 21.5% in untimely treated. Compared to untimely treated, the risk of death in TT patients was 46.6% higher (HR = 1.465; 95%CI: 1.381–1.555).

**Conclusions:**

LC survival is highly dependent on early diagnosis and adequate treatment. Time-to-treatment was longer than recommended for all treatment types but particularly for surgery. Overall TT results were paradoxical, as better survival was observed in patients untimely treated. The factors associated with TT were not possible to analyze and its impact on patient outcomes remains unclear. However, it is important to assess quality-of-care to improved LC management.

## Background

Lung cancer (LC) is a considerable public health problem because it incorporates a substantial burden in terms of morbidity and mortality [1]. In Portugal, LC was the first cause of cancer-related death and disability combined in 2019 [2]. Its management is a complex process, and the time between presentation of first symptoms, the required diagnosis procedures and treatment initiation involves a wide range of specialists [3].

The Institute of Medicine has identified timeliness-of-care as one of six dimensions of health-care quality [4]. In LC, less timely care may be associated with missed opportunities for cure or effective palliative treatment, and it probably contributes to the emotional distress of patients and their families [5,6]. Yet, the impact of treatment timeliness on clinical outcomes is unclear based on available evidence [6].

The goals for LC therapy are to achieve a cure where possible and/or palliative care, through symptom burden reduction and quality-of-life improvement. A variety of management approaches including surgery, radiation and systemic therapies may be used in LC, depending on histology, stage at diagnosis and patient’s fitness to tolerate therapy [6,7,8]. Standards for timely LC care have been established, through clinical opinion-based guidelines, by the British Thoracic Society (BTS) and the RAND Corporation [9].

Since timeliness-of-care is modifiable, evidence pertaining to its effect on survival is of particular importance [10]. While survival outcomes are frequently collected in registries, the impact of the disease and its treatment on patients’ quality-of-life is rarely assessed routinely, but quality of end-of-life care is an important consideration [7]. One of the measures proposed as indicator to assess aggressiveness of end-of-life care is starting a new chemotherapy regimen less than 30 days before death [11,12,13].

Our study aims to: 1) characterize LC patients registered in the regional population-based cancer registry for Southern Portugal and Madeira (ROR-SUL) from 2009–2014 and their treatment patterns; 2) analyze the quality of end-of-life care for LC patients and 3) estimate the time from diagnosis to treatment and its impact on survival. To our knowledge, this is the first study in Portugal to analyze the impact of quality of care on LC patient’s survival using population-based data.

## Materials and Methods

### Data and sources

Incident cases of invasive LC (ICD-10 code C34) [14] were obtained for the six-year period 2009 through 2014 from ROR-SUL, that covers 4,800,000 inhabitants (46% of country population) [15]. Patients were followed until the end of 2019. Informed consent was not necessary as data received were previously anonymized.

The following variables were analyzed: sex (male/female), age-groups (<40, 40–49, 50–59, 60–69, 70–79 and ≥80), tumor stage (I, II, III, IV), histologic types (non-small cell lung cancer [NSCLC]: non-small cell lung cancer not otherwise specified [NSCLC, NOS], adenocarcinoma [ADC], squamous cell carcinoma [SQCC], and Others; Small cell lung cancer [SCLC]; other and unspecified [OU]) [16]. Additionally, we analyzed treatment related variables: treatment received (Yes/No), treatment type (surgery (S)/radiotherapy (R)/systemic therapy (Sy)). The definition of treatment received should be interpreted as treatment information included in the registry. We cannot exclude the possibility that patients classified with “no treatment” had indeed received treatment that was not registered. Additionally, treatment definition excludes symptomatic and/or palliative treatment.

The quality indicator time-to-treatment (TTT) was defined as the period between diagnosis and initiation of first treatment. Timely treatment (TT) was defined as treatment initiation within the recommended thresholds specified in the BTS [17] for the specific treatment types (surgery ≤ eight weeks (56 days), radiotherapy ≤ four weeks (28 days) and systemic treatment ≤ three weeks (21 days)) and in the RAND Corporation [18] for all treatments ≤ six weeks (42 days). Untimely treatment was defined as treatment initiation above the recommended thresholds.

### Statistical methods

We performed descriptive analyses of total LC cases from 2009–2014, presenting proportions for categorical variables and mean with standard deviation and medians with inter-quartile range (IQR) for continuous variables. Median TTT was calculated overall, by treatment type and per stage. A chi-squared test was used to assess the association between patient characteristics and presence of treatment and TT. After descriptive analyses, we estimated the impact of treatment and TT in the total population and by disease stage on five-year overall survival (OS) using the Kaplan-Meier method, compared by log-rank tests. The duration of OS was calculated from the date of diagnosis until death or date of last follow-up. The association between OS and treatment and OS and TT was examined with a Cox multiple regression model with risk ratios measured by Hazard Ratio (HR). Through Cox multiple regression, we determined the HR of death associated with treatment and TT, adjusted for the effect of all other variables in the equation [19]. The model included the following variables: sex, age, histology and stage.

The level of statistical significance for all comparisons was 0.05. Data was analyzed using Statistical Package for Social Services (IBM SPSS Statistics for Windows, version 26.0, Armonk, NY).

## Results

Overall, there were 11,308 LC cases diagnosed, of which 8,557 (75.7%) male and 2,751 (24.3%) female. Two cases were excluded from the survival analysis due to absence of survival information and 227 were lost-to follow-up. Mean age at diagnosis was 66.15 years old (11.317) and median age 67 years old (IQR: 58–75). The number of LC cases increased with age, with the age groups of 60–69 and 70–79 years old presenting the highest number of cases (Table 1).

**Table 1:** Differences in treatment receipt and type of treatment by sex, age group, histology and stage, in ROR-SUL, 2009–2014.


	N (PATIENTS)	TREATMENT, N (%)			TYPE OF TREATMENT, N (%)					
		
	TOTAL	YES	NO	p-VALUE	S (%)	p-VALUE	R (%)	p-VALUE	Sy (%)	p-VALUE

**Sex, N (%)**	11,308 (100)	6,976 (61.7)	4,332 (38.3)	<0.0001	**2,326 (20.6)**	**<0.0001**	2,855 (25.2)	0.235	4,412 (39.0)	0.880

Male	8,557 (75.7)	5,200 (60.8)	3.357 (39.2)		**1.635 (19.1)**		2,184 (25.5)		3,342 (39.1)	

Female	2,751 (24.3)	1.776 (64.6)	975 (35.4)		**691 (25.1)**		671 (24.4)		1,070 (38.9)	

**Age group, N (%)**	11,308 (100)	6,976 (61.7)	4,332 (38.3)	<0.0001	**2,326 (20.6)**	**<0.0001**	**2,855 (25.2)**	**<0.0001**	**4,412 (39.0)**	**<0.0001**

<40 years	130 (1.2)	99 (76.2)	31 (23.8)		**49 (37.7)**		**41 (31.5)**		**47 (36.2)**	

40–49 years	768 (6.8)	566 (73.7)	202 (26.3)		**200 (26.0)**		**294 (38.3)**		**359 (46.7)**	

50–59 years	2,240 (19.8)	1,592 (71.1)	648 (28.9)		**520 (23.2)**		**744 (33.2)**		**1,111 (49.6)**	

60–69 years	3,521 (31.1)	2,341 (66.5)	1,180 (33.5)		**808 (22.9)**		**937 (26.6)**		**1,540 (43.7)**	

70–79 years	3,315 (29.3)	1,899 (57.3)	1,416 (42.7)		**633 (19.1)**		**668 (20.2)**		**1,119 (33.8)**	

>80 years	1,334 (11.8)	479 (35.9)	855 (64.1)		**116 (8.7)**		**171 (12.8)**		**236 (17.7)**	

**Year of Diagnosis N (%)**	11,308 (100)	6,976 (61.7)	4,332 (38.3)	<0.0001	**2,326 (20.6)**	**<0.0001**	**2,855 (25.2)**	**<0.0001**	**4,412 (39.0)**	**<0.0001**

2009	1,801 (15.9)	1,028 (57.1)	773 (42.9)		**439 (24.4)**		**417 (23.2)**		**461 (25.6)**	

2010	1,815 (16.0)	939 (51.7)	876 (48.3)		**357 (19.7)**		**373 (20.6)**		**497 (27.4)**	

2011	1,815 (16.0)	1,012 (55.8)	803 (44.2)		**319 (17.6)**		**426 (23.5)**		**598 (32.9)**	

2012	2,023 (17.9)	1,195 (59.1)	828 (40.9)		**410 (20.3)**		**469 (23.2)**		**758 (37.5)**	

2013	1,937 (17.1)	1.393 (71.9)	544 (28.1)		**419 (21.6)**		**594 (30.7)**		**1,056 (54.5)**	

2014	1,917 (17.0)	1,409 (73.5)	508 (26.5)		**382 (19.9)**		**576 (30.0)**		**1,042 (54.4)**	

**Histology, N (%)**	11,308 (100)	6,976 (61.7)	4,332 (38.3)	<0.0001	**2,326 (20.6)**	**<0.0001**	**2,855 (25.2)**	**<0.0001**	**4,412 (39.0)**	**<0.0001**

NSCLC										

ADC	5,095 (45.1)	3,409 (66.9)	1,686 (33.1)		**1,302 (25.6)**		**1,353 (26.6)**		**2,136 (41.9)**	

SQCC	2,423 (21.4)	1,493 (61.6)	930 (38.4)		**480 (19.8)**		**623 (25.7)**		**992 (40.9)**	

Others^1^	740 (6.5)	496 (67.0)	244 (33.0)		**311 (42.0)**		**129 (17.4)**		**203 (27.4)**	

NSCLC NOS	847 (7.5)	506 (59.7)	341 (40.3)		**73 (8.6)**		**262 (30.9)**		**338 (39.9)**	

SCLC	1,164 (10.3)	680 (58.4)	484 (41.6)		**94 (8.1)**		**309 (26.5)**		**509 (43.7)**	

OU	1,039 (9.2)	392 (37.7)	647 (62.3)		**66 (6.4)**		**179 (17.2)**		**234 (22.5)**	

**Stage, N (%)**	8,531 (75.4)	6,171 (72.3)	2,360 (27.7)	<0.0001	**2,326 (20.6)**	**<0.0001**	**2,855 (25.2)**	**<0.0001**	**4,412 (39.0)**	**<0.0001**

I	1,187 (13.9)	1,045 (88.0)	142 (12.0)		**893 (75.2)**		**125 (10.5)**		**214 (18.0)**	

II	476 (5.6)	378 (79.4)	98 (20.6)		**271 (56.9)**		**91 (19.1)**		**241 (50.6)**	

III	1,877 (22.06)	1,449 (77.2)	428 (22.8)		**325 (17.3)**		**797 (42.5)**		**1,187 (63.2)**	

IV	4,491 (58.5)	3,299 (66.1)	1,692 (33.9)		**387(7.8)**		**1,707 (34.2)**		**2,450(49.1)**	

Unknown	2,777 (24.6)	805 (29.0)	1,972 (71.0)		**450 (16.2)**		**135 (4.9)**		**320 (11.5)**	


ADC = Adenocarcinoma; SQCC = Squamous Cell Carcinoma; NSCLC NOS = Non-Small Cell Lung Cancer Not Otherwise Specified; SCLC = Small Cell Lung Cancer; OU = Other Unspecified; ^1^ Others: include large cell carcinoma, mixed carcinoma, neuroendocrine lung cancer, and others.

From all cases, 8,531 (75.4%) had information on stage; most presented with locally advanced (N = 1,877, 22.0%) or metastatic disease (N = 4,991, 58.5%). During the period in analysis although stages III and IV accounted for approximately 80% of cases, we observed a marked decline in the percentage of unknown stage, particularly in the last two years, from 36.1% in 2009 to 5.6% in 2013 and 7.6% in 2014. NSCLC accounted for 80.5% of cases of which most common type was ADC (45.1%), followed by SQCC (21.4%). SCLC represented 10.3% of cases and OU 9.2%.

Overall, 6,976 (61.7%) received treatment and 4,332 (38.3%) received no treatment. Among treated patients, 2,326 (33.3%) received surgery, 2,855 (40.9%) radiotherapy and 4,412 (63.2%) systemic treatment (Table 1). Additional treatment type details can be found in Supplement I.

Women received treatment more often than men (64.6% vs. 60.8%, p < 0.0001), and the same was true for surgery (25.1% vs. 19.1%, p < 0.0001). No sex differences were observed for radiotherapy or systemic treatment. Treatment rate decreased with increasing age ranging from 76.2% (<40) to 35.9% (≥80). We verified the same pattern for surgery with 37.7% (<40) and 8.7% (≥80), and approximate for radiotherapy and systemic treatment. Across the six-year period we observed a 28.7% increase in the treatment rate, evident in radiotherapy but mostly in chemotherapy. Highest treatment rates were observed for ADC (66.9%) and Others (67%), followed by SQCC (61.6%), and the lowest rate was found for OU (37.7%). Overall, 72.3% patients with defined stage received treatment compared to 24.6% patients with unknown stage. Treatment rate patterns decreased with increasing stage, 88% (stage I), 79.4% (stage II), 77.2% (stage III) and 66.1% (stage IV). The same was observed for surgical treatment ranging from 75.2% stage I patients treated vs. 7.8% Stage IV, but an almost opposite trend for radiotherapy and systemic treatment (Table 1).

From the 6,975 patients treated, 871 were excluded from the TT analysis (156 due to treatment initiation before registered date of diagnosis and 715 with first treatment matching date of diagnosis). Patients with treatment initiation on the date of diagnosis might be explained by a diagnosis done during surgery and may have specific conditions different from the other patients and introduce bias, or lack the registry information due to limited access to clinical information by the registers.

The overall median TTT was 49 days (IQR: 28–88) and 43.3% received TT. Considering TT per treatment type, median TTT were 64 days (IQR: 29–112) to surgery, 46 days (IQR: 27–82) to radiotherapy and 45 days (IQR: 28–77) to systemic therapy. Timely surgery was received by 44.7% patients, timely radiotherapy by 28% and timely systemic treatment by 16.8%.

Women received TT more frequently than men, 43.8% versus 40.6% (p = 0.025). Younger patients (<40) received TT more often than elderly, 56.8% versus 37.3% (70–79 years old) and versus 34.4% (≥80) (p < 0.0001). Patients in earlier stages had lower rates of TT compared to more advanced, 24.7% in stage I, 27.4% stage II, 35.3% stage III and 51.3% stage IV (p < 0.0001). According to the previous observation, median TTT improved with more severe stages namely 80 days for stage I, 69.5 days stage II, 55 days stage III and 42 days for stage IV (Table 1).

Post-operative (PO) mortality at 30 days was 0.9% and increased to 1.5% and 2% at 60 and 90-days, respectively. An improvement in 30-day PO mortality was observed throughout the six-year period ranging from 1.8% in 2009 to 0.2% in 2014 (p < 0.0001), reflecting an 88.9% improvement. In what concerns the aggressiveness-of-care measure, driven by time from first systemic treatment initiation to death in less than 30 days, we observed that it occurred in 5.5% of patients. No significant differences were found across the period in analysis (p = 0.069).

Median five-year OS was 279 days (95%CI: 269.1–288.9) and survival proportion 14.9%. Median survival times and OS for treated and untreated patients were 430 days and 86 days, 19.6% and 7.1%, respectively (Table 2). Treatment impact showed higher differences in early stages, 64.1% versus 32.9% (stage I) and 44% versus 12% (stage II) survival for treated compared to untreated patients. Contrary to treatment, TT showed a negative impact on survival, 11.3% versus 21.5%. The median survival was 280 days among those TT, and 553 days among those untimely treated (p < 0.0001). TT had no impact in stages I/II survival but in stages III/IV patients without TT had better survival, 14.9% versus 17.3% and 2.6% versus 4.5%, respectively.

**Table 2:** Five-year overall survival related to treatment received, type of treatment and timely treatment, per sex and stage, ROR-SUL, 2009–2014.


	MEDIAN SURVIVAL IN DAYS (95%CI)				KAPLAN-MEIER OS (%) (95%CI)		

**Treatment received**							

	All	Yes	No	p value	All	Yes	No

All	279 (269.1–188.9)	430 (413.9–446.1)	86 (79.6–92.4)	p < 0.0001	14.9 (14.3–15.5)	19.6 (18.6–20.6)	7.1 (6.3–7.9)

Stage				p < 0.0001			

I	n.r.	n.r.	600 (313.5–886.5)		60.4 (57.7–63.1)	64.1 (61.2–67.0)	32.9 (25.1–40.7)

II	1,086 (861.6–1310.4)	1,377 (1074.6–1679.4)	182 (64.6–299.4)		37.4 (33.1–41.7)	44.0 (38.9–49.1)	12.0 (5.5–18.5)

III	424 (397.7–450.3)	533 (498.7–567.3)	115 (90.3–139.7)		14.1 (12.5–15.7)	16.9 (14.9–18.9)	4.6 (2.6–6.6)

IV	171 (161.8–180.2)	264 (252.5–275.5)	51 (46.8–55.2)		2.8 (2.4–3.2)	3.5 (2.9–4.1)	1.6 (1.0–2.2)

**Timely Treatment** ^1^							

	All	Yes	No	p value	All	Yes	No

All	411 (395.8–426.1)	280 (264.0–296.0)	553 (426.0–580.0)	p < 0.0001	17.1 (16.1–18.1)	11.3 (10.1–12.5)	21.5 (20.1–22.9)

Stage				p < 0.0001			

I	n.r.	n.r.	n.r.		63.3 (59.8–66.8)	63.9 (56.8–71.0)	63.2 (59.1–67.3)

II	1,363 (1046.4–1679.5)	1,363 (–)	1,370 (1017.6–1722.4)		43.7 (38.4–49.0)	45.1 (35.1–55.1)	43.1 (36.8–49.4)

III	526 (491.9–560.1)	426 (389.7–462.3)	586 (545.8–626.2)		16.5 (14.5–18.5)	14.9 (11.8–18.0)	17.3 (14.8–19.8)

IV	269 (257.6–280.4)	204 (188.0–220.0)	341 (319.5–362.5)		3.5 (2.9–4.1)	2.6 (1.8–3.4)	4.5 (3.5–5.5)

**Timely Treatment per treatment type**							

	All	Yes	No	p value	All	Yes	No

Surgery^2^	n.r.	1,374 (–)	n.r.	p < 0.0001	51.7 (49.2–54.2)	46.5 (42.8–50.2)	55.9 (52.6–59.2)

Radiotherapy^3^	358 (339.2–376.8)	229 (206.1–251.9)	424 (398.3–449.6)	p < 0.0001	9.2 (8.0–10.4)	5.9 (4.1–7.7)	10.6 (9.2–12.0)

Systemic^4^	395 (338.8–409.2)	284 (254.5–313.5)	420 (403.9–436.1)	p < 0.0001	11.4 (10.4–12.4)	7.9 (5.9–9.9)	12.1 (10.9–13.3)

**Timely Surgery** ^2^							

Stage				p < 0.0001			

I	n.r.	n.r.	n.r.		74.5 (71.0–78.0)	76.7 (70.8–82.6)	73.3 (68.8–77.8)

II	n.r.	n.r.	n.r.		57.3 (50.8–63.8)	62.2 (52.4–72.0)	54.1 (45.9–62.3)

III	1,309 (1010.4–1607.6)	1,374 (776.7–1971.3)	1,240 (890.1–1589.9)		41.0 (35.3–46.7)	44.0 (34.8–53.2)	39.1 (31.8–46.4)

IV	391 (322.8–459.2)	347 (290.1–403.9)	578 (514.3–641.7)		10.9 (7.2–14.6)	10.9 (6.6–15.2)	11.3 (3.5–19.1)


^1^ Timely treatment according to RAND corporation guidelines (any type of treatment up to 6 weeks), 871 treated patients excluded from the analysis considering time from diagnosis to treatment was ≤0 (<0 – n = 156; 0 – n = 715); ^2^ Timely treatment according to BTS guidelines (surgery up to 8 weeks), 824 treated patients excluded from the analysis considering time from diagnosis to treatment was ≤0 (<0 – n = 135; 0 – n = 689), ^3^ Timely treatment according to BTS guidelines (radiotherapy up to 4 weeks), 101 treated patients excluded from the analysis considering time from diagnosis to treatment was ≤0 (<0 – n = 32; 0 – n = 69); ^4^ Timely treatment according to BTS guidelines (systemic therapy up to 3 weeks), 105 treated patients excluded from the analysis considering time from diagnosis to treatment was ≤0 (<0 – n = 31; 0 – n = 74); n.r. = not reached.

TT was also associated with worse survival for all treatment types. This impact was less pronounced for surgery (46.5% vs. 55.9%) than radiotherapy (5.9% vs. 10.6%) or systemic treatment (7.9% vs. 12.1%).

A deeper analysis conducted on the impact of timely surgery according to stage showed a positive impact of TT for earlier stages and advanced localized disease: 76.7% versus 73.3% (stage I), 62.2% versus 54.1% (stage II), 44.0% versus 39.0% (stage III), but a slight inversion for metastatic disease, 10.9% versus 11.3% (Table 2).

In the Cox multivariable model, the adjusted mortality risk was significantly higher in untreated patients, relative to those treated (HR = 2.420; 95%CI: 2.293–2.553, p < 0.0001). However, TT had a negative effect on survival. The adjusted mortality risk for patients TT was 46.5% higher, referenced to untimely treated (HR = 1.465; 95%CI: 1.381–1.555, p < 0.0001).

## Discussion

In the regions covered by ROR-SUL, patient’s characterization by sex, age, histology and stage was similar to what was previously reported for the whole country, but with better staging information available (75.4% vs. 53.1%) [20].

Treatment rate difference observed among men and women was driven by surgery. In accordance with another publication, surgery rate was 31% higher in women compared to men (25.1% vs. 19.1%, p < 0.0001) [21]. This can be partially explained by the higher stage I rate in women compared to men (20.6% vs. 13.1%) and more stage I women receiving surgery (78.7% vs. 73.4%). Although reasons for these sex discrepancies are unclear, higher 90-day mortality reported in men strongly suggests they are poorer surgical candidates [21].

Elderly patients received curative surgery less frequently and although this can be due to concerns with increased frailty and treatment complications, some studies reported that this happened independently from co-morbidities [22]. The absence of histologic differentiation is linked with lower treatment rates as it is harder to select the most appropriate treatment for those patients [23].

Treatment recommendations for LC vary significantly upon stage. Although surgery is the initial treatment in operable stages I through IIIA, patients with non-resectable or borderline resectable LC at initial presentation receive neo-adjuvant (or definitive) chemoradiation to prolong survival [23]. Definitive chemotherapy is considered appropriate for stage IV patients that have good performance status [23]. Overall, our results reflect the differences expected for the different treatment types per stage. The proportions of stage I (75.2%) and II (56.9%) patients who receive surgery positioned well within the quality indicator goal >50% found in the literature [24]. Regarding the goal >60% proposed for the proportion of patients in advanced stage that receive chemotherapy this was observed for stage III patients (63.2%) but nor for stage IV (49.1%) [24], but this could be eventually linked with patients’ performance status.

Timely LC care metrics vary between healthcare organizations, but it is generally accepted that there are justified reasons for delay for a specific proportion of patients. These reasons can include the complexity of diagnostic procedures, presence of co-morbidities and poor performance status and a patient’s decision to have breaks between diagnosis procedures or before treatment initiation [25]. Our analysis showed that only 43.3% of patients received TT, with median time-to-treatment of 49 days (IQR: 28–88) worse than in other publications that reported TT rates of 77.5% (9) and 80% [23]. As previously reported [8,9,26], surgery had the longest median TTT (64 days, IQR: 29–112) and only 44.7% of patients received TT, which can be due to the longest diagnostic procedures that reflect the extra time needed to refer patients to thoracic surgeon for additional treatment considerations [9]. A multidisciplinary team approach involving both surgeons and oncologist in the care process may help to minimize such delay [9]. Radiotherapy and chemotherapy presented lower median TTT than surgery probably due to the influence severity of disease might have on the speed of medical decision-making [9,26], nevertheless TT rates were lower, 28% and 16.8% respectively.

Treatment-related mortality and treatment intensity near the end-of-life are markers of the quality and safety of the care provided and treatment should only be undertaken in patients who may benefit from it [24,27]. A measure considered is the percentage of patients who received chemotherapy during the last 30-days of life and data reported presents high variability (6.4 to >50%) [28]. The wide differences observed across countries (5.7% in Norway, 5.9% in Canada, 12.1% in USA and >16% in the Netherlands and Germany can be explained by the different healthcare systems, payment organizations and cultural and societal considerations [28,29]. In our study, death <30-days after first chemotherapy treatment occurred in 5.5% of cases. The difference between our results and the others reported is likely linked with the broader definition of chemotherapy regimen (e.g., existence of chemotherapy instead of initiation).

Post-operative (PO) mortality rates are an important outcome measure to help guide clinicians and patients’ expectations [30]. There is evidence suggesting that 30-day mortality rate underestimates a patient’s true risk of death following LC resection, and that the risk of death at 90-days is almost twice higher [30]. Our analyses support this evidence with PO mortality rates of 0.9% (30 days), 1.5% (60 days) and 2% (90 days). The 30-day PO mortality rate observed in our data compares well with other countries reports, 2.0% in Belgium [27], 1.1% in Italy [31], 2.1% in USA [32], and 3.6% in Denmark [33].

Our study analyzed lung cancer OS and the impact of treatment and TT on patient outcomes for the total population and stratified by stage. In this population OS was slightly higher than previously reported for the whole country 14.9% vs. 13.6% [34], what might be attributed to the longer period analyzed (2009–2014 vs. 2009–2011) that likely benefited from the improvement in therapeutic options like targeted therapies for specific mutations and immune-checkpoint inhibitors. As expected, treatment had a protective effect on survival. The reasons for treatment absence were not possible to assess as information on patient performance status and presence of co-morbidities were not available. Nevertheless, considering the higher incidence of LC in older patients, that close to 80% had locally advanced or metastatic disease and consequently more symptomatic and severe, it’s likely that in the majority of these patients, treatment was no longer viable. Nonetheless, it is important to highlight that these patients might have received symptomatic and/or palliative treatment, therapies that are not considered for inclusion in the registry, or even treatment not registered.

Identifying LC patients in an early stage, when more likely to be suitable for potentially curative surgery, is the key to improve survival [3], and this was evident in our study. Stage progression is associated with decreased surgical cure rates and poorer OS [35].

Regarding the impact of TT in survival our results are counterintuitive as we observe worse survival in patients that receive TT compared with delayed treatment 11.3 % versus 21.5%. Although timeliness is an important dimension of quality-of-care for patients with LC [8], and may contribute to patients’ quality-of-life and emotional well-being, it remains unclear if timely care also improves patient outcomes [36].

A 2009 systematic review on the timeliness of LC care concluded that reported time-to-treatment often exceeded guidelines recommendations [36]. The impact of timeliness of care on outcomes was variable, with three studies reporting better survival with more timely care, eight studies reporting no association, and four studies reporting paradoxically poorer survival with more timely care [35,36]. Results of more recent studies have also been mixed [35].

Several studies reported this phenomenon commonly called “waiting time paradox” in which more timely care was associated with worse outcomes [1,5,8,9,10,23,26]. These paradoxical results are likely explained by residual confounding by severity, which means that patients with more advanced or aggressive disease, that are more symptomatic, are more likely to receive timely treatment but less likely to survive than patients in earlier stages of disease, due to the severity of their condition at presentation [1,5,10,23]. Although timely care was not always associated with better prognosis, any delays in treatment not related with a proper time allocation for optimal assessments, should be avoided, as it may increase the risk of disease progression and psychological stress in patients [9,10].

TT impact on survival per stage presents conflicting results with no substantial differences observed in stages I and II but better survival for stages III (+16.1%) and IV (+73.1%) for patients receiving untimely versus TT. Similar findings were reported by Nadpara and colleagues that observed non-significantly better survival outcomes with timely care in patients with early-stage disease (Stage I/II). Among patients with late-stage disease (Stage III/IV), survival outcomes were found to be significantly worse among those receiving TT [9]. A deeper analysis on timely surgery’s impact on the different disease stages showed a positive effect of TT on survival for stages I (+ 4.6%), II (+15%), III (+12.5%) and a negative effect for stage IV (-3.5%), what reinforces the importance of earlier diagnosis and faster treatment.

The main strength of our study lies in the population-based approach that covers almost half of the country population. Additionally, to our knowledge this is the first study to date to examine the impact of treatment timeliness in LC survival in Portugal. The results suggest that there is room for improvement in TT rates although it is difficult to know if these results were related with patient specific conditions, procedures for better histologic characterization or staging requirements or, on the contrary, due to avoidable delays. Good results are suggested in what concerns short-term post-operative mortality or end-of-life chemotherapy exposure. Quality of end-of-life care is an important consideration in LC although it is not clear whether any treatment near the end-of-life should be considered a marker of poor quality as some procedures or therapies may be effective in alleviating distressful symptoms [7].

Our study has limitations that need to be highlighted. The population-based registries can have poorly classified, incomplete or missing data related to treatment; therefore, we cannot exclude the possibility that patients classified as not treated had received treatment that was not registered. The high level of missing stage information (24.3%) does not allow these patients to be included in some of the analysis. The lack of clinical variables such as performance status, a strong predictor of survival in LC [7], and the absence of co-morbidities information often complicate data interpretation, particularly when addressing treatment specificities and timeliness that are largely impacted by these factors. No information was available on the operability of tumors and other findings than can dictate the appropriate treatment, including the absence of data regarding the existence of a multidisciplinary team approach and its impact on the time from diagnosis to treatment and treatment timeliness.

While it remains unclear whether more timely care improves outcomes in LC, improving timeliness is important independent of its ultimate effect on survival. Patients expect, deserve and appreciate care that is timely, safe and effective [36]. Assessing quality of care is one of the first steps in ultimately improving patients’ health outcomes as it provides a baseline of the rate of current health practices [37].

Lung cancers still have a poor overall prognosis, mainly related to the advanced stage at diagnosis. The importance of early-stage diagnosis, linked with the higher rates of curative treatment provides powerful evidence of the positive impact that could come from the implementation of screening programs for high-risk LC patients. Nevertheless, a screening program would require a smooth process to manage patients with suspicion of lung cancer to out rule potential false positives and/or secure a prompt diagnosis and adequate and timely treatment initiation. This could be enabled through the implementation of multidisciplinary organized care in all centers to improve the entire management process incorporating the required diagnostic procedures and specialized consultations to support treatment decisions, and setting clear quality indicators for TTT and TT. Additionally, these quality indicator results obtained from the multidisciplinary team approach should be continuously monitored versus recommended guidelines to enhance systematic improvement, and contribute to reduce patient anxiety due to avoidable delays and improve satisfaction with the care provided.

## Conclusions

Our study contributed to the evidence that LC survival is highly dependent on early diagnosis and adequate treatment. Time-to-treatment was longer than recommended in guidelines for all treatment types but particularly for surgery. The overall results in treatment timeliness were paradoxical as better survival was observed in patients untimely treated. The factors associated with TT were not possible to analyze and it remains unclear if timely care will improve patient outcomes.

However, assessing quality of care is an important step towards healthcare practice improvement for LC patients.

## Appendix

**Supplement I:** Distribution of treatment type per stage at diagnosis.

	I N (%)	II N (%)	III N (%)	IV N (%)	UNKNOWN N (%)	p-VALUE

	**1,188 (13.9)**	**476 (5.6)**	**1,877 (22.0)**	**4,991 (58.5)**	**2,777 (24.6)**	

**Type of treatment, N (%)**						

Any treatment	1,045 (88.0)	387 (79.4)	1,449 (77.2)	3,299 (66.1)	805 (29.0)	<0.0001

No treatment	143 (12.0)	98 (20.6)	428 (22.8)	1,692 (33.9)	1,972 (71.0)	<0.0001

Surgery (S) only	743 (62.5)	97 (20.4)	59 (3.1)	144 (2.9)	410 (14.8)	<0.0001

Radiation (R) only	76 (6.4)	34 (7.1)	176 (9.4)	637 (12.8)	70 (2.8)	<0.0001

Systemic (Sy) only	49 (4.1)	49 (10.3)	483 (25.7)	1,390 (27.9)	233 (8.4)	<0.0001

S + R+ Sy	10 (0.8)	27 (5.7)	129 (6.9)	117 (2.3)	8 (0.3)	<0.0001

S + R	12 (1.0)	6 (1.3)	27 (1.4)	68 (1.4)	5 (0.2)	0.764

S + Sy	128 (10.8)	141 (29.6)	110 (5.9)	58 (1.2)	27 (1.0)	<0.0001

R + Sy	27 (2.3)	24 (5.0)	465 (24.8)	885 (17.7)	52 (1.9)	<0.0001

S total	893 (75.2)	271 (56.9)	325 (17.3)	387 (7.8)	450 (16.2)	<0.0001

R total	125 (10.5)	91 (19.1)	797 (42.5)	1,707 (34.2)	135 (4.9)	<0.0001

Sy total	214 (18.0)	241 (50.6)	1,187 (63.2)	2,450 (49.1)	320 (11.5)	<0.0001
